# Comparative Outcomes of Direct Versus Mesh Repair and Timing of Repair for Traumatic Abdominal Wall Hernias: A Systematic Review and Meta‐Analysis

**DOI:** 10.1111/ans.70265

**Published:** 2025-07-25

**Authors:** Khang Duy Ricky Le, Su Jin Lee, Rose Shakerian, Benjamin P. T. Loveday, David Read

**Affiliations:** ^1^ Trauma Service The Royal Melbourne Hospital, Parkville Melbourne Victoria Australia; ^2^ Department of Surgery The University of Melbourne, Parkville Melbourne Victoria Australia

**Keywords:** recurrence, timing of repair, trauma laparotomy, traumatic abdominal wall hernia

## Abstract

**Introduction:**

Traumatic abdominal wall hernia (TAWH) refers to the disruption of the muscular layers of the abdominal wall following blunt traumatic injury. There is a lack of consensus in the management of TAWH, particularly when trauma laparotomy for concurrent visceral injury is required. This systematic review and meta‐analysis aims to evaluate recurrence outcomes with repair technique (mesh vs. direct suture repair) and timing of repair (acute vs. elective) with and without trauma laparotomy.

**Methodology:**

A comprehensive search was conducted on Medline, Embase, and Cochrane central databases. Keywords related to traumatic abdominal wall hernia, acute and elective treatment, and timing of repair were used to identify relevant articles.

**Results:**

A total of 26 studies involving 432 patients were included. There was reduced recurrence with mesh compared to direct suture repair in patients with TAWH who did not require trauma laparotomy (OR 0.20, 95% CI 0.05–0.82, *p* = 0.02), although there was no difference in recurrence between acute versus elective repair in this cohort. There was no difference between mesh and direct suture repair for the management of TAWH in patients requiring trauma laparotomy.

**Conclusion:**

This systematic review and meta‐analysis identified evidence of reduced recurrence with mesh compared to direct suture repair for a patient with TAWH who did not require trauma laparotomy. There was insufficient evidence of superiority for mesh compared to direct suture repair in trauma laparotomy settings, nor timing of repair in non‐trauma laparotomy settings. The strength of the conclusions is limited by the risk of bias in included studies and their heterogeneity.

## Graphical abstract

This systematic review and meta‐analysis seeks to evaluate the current evidence to characterize whether the timing of repair (acute or elective) and the mode of repair (mesh or direct suture) influence recurrence outcomes in patients with traumatic abdominal wall hernia in laparotomy and non‐laparotomy settings.

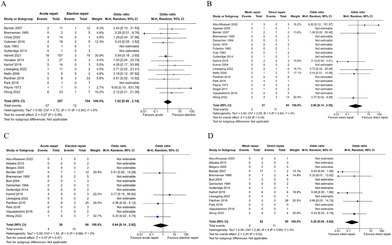

## 1 Introduction

Traumatic abdominal wall hernia (TAWH) refers to disruption of the abdominal wall muscular layers following blunt traumatic injury [1]. These injuries are uncommon, present in less than 1% of admissions secondary to blunt abdominal trauma [2, 3]. In Australia, there is limited reporting of TAWH epidemiology, with two retrospective observational studies from major trauma centres identifying an incidence of 0.5% and 1.54% of f patients presenting with blunt abdominal trauma, with the former study identifying only 47 patients within a 9‐year period [4, 5]. Importantly, TAWHs occur in the context of significant force, such as motor vehicle crashes, pedestrian injuries, and high falls, and are often associated with multiple other injuries from both intra‐ and extra‐abdominal sites. As such, up to a third of patients with a TAWH require a laparotomy for concurrent intra‐abdominal injury [2, 3].

TAWHs present in a range of contexts and may be classified using various methods, with the most common being the classification system proposed by Dennis et al. [1]. In this system, TAWHs are graded between I and VI; where I refers to subcutaneous tissue contusion, II referring to abdominal wall haematoma, III referring to single abdominal wall muscle disruption and scores above IV referring to complete abdominal wall muscle disruption with or without herniation of abdominal contents or evisceration [1]. The of management TAWH is influenced by the types of concurrent injury, patient factors, surgeon‐specific skills, and health system factors. There is a lack of consensus in the management of TAWHs, perhaps fostered by this variability of presentation and their relative rarity. The two common clinical dilemmas that face the treating surgeon are the timing of repair, loosely categorized as early on during the index admission or delayed several months after discharge, and the method of repair, particularly considering the role of mesh compared to direct suture repair. Some observational studies have suggested delayed repair may be preferable, demonstrating an increased recurrence rate with acute or immediate repair [5, 6, 7]. Conversely, other observational studies have shown no association between recurrence rates and the timing of repair [8]. Although generally the use of mesh is considered to lower hernia recurrence rates, many TAWH patients have a contaminated field due to concurrent enteric injury. Furthermore, physiological responses to trauma have secondary effects on wound healing, coagulation, and response to infection which may impact hernia management and outcomes [9, 10, 11]. At present, there is divided opinion regarding immediate versus delayed repair and the use of mesh versus direct suture repair in these populations. Furthermore, when mesh is favored, the choice between synthetic versus biologic mesh and the influence of this on recurrence outcomes is poorly defined.

Given this, there are two clinical scenarios where management remains unresolved. The first is whether, in patients with a TAWH who do not undergo a trauma laparotomy for other intra‐abdominal injury, the timing of repair (acute or delayed) or mode of repair (direct versus mesh) influences hernia recurrence rate. The second is whether, in patients with a TAWH who do undergo an urgent trauma laparotomy, mesh or direct suture repair differs in terms of hernia recurrence rate. Therefore, this systematic review and meta‐analysis aimed to evaluate the current literature regarding the relationship between TAWH recurrence rates and two questions: [1] Repair technique (mesh vs. direct suture repair) and [2] timing of repair (acute vs. elective). This information may assist in providing an evidence base for the management of TAWH.

## 2 Methods

### 2.1 Search Strategy

This review was performed according to the Preferred Reporting Items for Systematic Review and Meta‐Analyses (PRISMA) guidelines. The review protocol was prospectively registered in the PROSPERO database (PROSPERO ID: CRD42023463259).

A literature search was conducted on Medline, Embase, and Cochrane central databases on 5 September 2023. Additional relevant articles from the reference lists of captured studies were identified and included where relevant. The search strategy combined medical subject headings (MeSH) and keywords related to traumatic abdominal wall hernia, acute and elective treatment, and timing of repair. Truncations and Boolean operators were used during the search to find all relevant articles.

### 2.2 Eligibility Criteria

Peer‐reviewed publications available in full text and in English language, which evaluated the recurrence of hernia in patients with TAWH, were considered for this review.

Articles were included if they: (1) were original and peer‐reviewed randomized‐controlled trials, non‐randomized trials, or observational studies; (2) analyzed data from adults aged ≥ 18 years with traumatic abdominal wall hernia; and (3) evaluated recurrence of hernia following operative intervention factoring in timing of repair, use of mesh, and type of surgery.

Articles were excluded if they were the following study types: reviews, meta‐analyses, conference papers, letters and editorials, commentaries, or were non‐human trials. Articles were excluded if they had incomplete data, evaluated abdominal wall hernias that were not a result of traumatic injury, or did not explore outcomes relevant to this review.

### 2.3 Literature Screening

Screening of title and abstract was performed by two independent investigators (KL, SL). Titles and abstracts that did not provide sufficient information progressed to full‐text analysis. The same two investigators independently performed a full‐text analysis based on eligibility criteria. Disagreement during this process was resolved by consensus.

### 2.4 Endpoints

The primary outcome of interest was the development of hernia recurrence confirmed radiologically or clinically. This was considered in two contexts: patients who underwent TAWH repair during trauma laparotomy, with a comparison between mesh and direct suture repair, and patients who did not undergo trauma laparotomy and had repair of their TAWH, with a comparison between acute and elective repair. Acute repair was defined as repair during the index trauma admission. Elective repair was defined as repair that occurred following discharge from the index trauma admission.

### 2.5 Data Extraction

Data extracted included study name, first author, design, and country; year of publication; demographic and baseline data of study cohorts (age, sex, total number of patients, site of TAWH), intervention data (intervention performed, timing of repair) and outcome data (recurrence of TAWH, time to recurrence). Where additional data were required, corresponding authors were contacted to request unpublished data for demographic factors and outcomes of interest.

### 2.6 Quality Assessment

Articles were assessed for methodological quality using the Risk Of Bias in Non‐randomised Studies of Interventions (ROBINS‐I) tool by two independent investigators (KL, SL) [11, 12]. Quality of individual parameters was considered on the spectrum of low, moderate, serious, and critical risk of bias. An overall risk of bias was then determined. Disagreement during this process was resolved by consensus. Publication bias was assessed with a funnel plot as part of the statistical analysis of included articles using IBM SPSS Statistics software version 29 (International Business Machines Corporation [IBM], Armonk, New York, USA).

### 2.7 Statistical Analysis

Statistics analysis was performed utilizing Review Manager 5.4 (RevMan 5.4, Cochrane Collaboration, London, United Kingdom). Odds ratios (OR) and their 95% confidence intervals (95% CI) of recurrence were calculated. Heterogeneity between studies was evaluated with the Higgins *I* ^2^ test [7, 8]. Values of *I* ^2^ at 25%, 50%, and 75% were graded as low, moderate, and high heterogeneity, respectively. A fixed‐effects model was used if substantial heterogeneity was absent, and a random‐effects model was used if substantial heterogeneity was found. A *p* value of < 0.05 was considered to be statistically significant. To determine sources of heterogeneity, subgroup analyses where relevant were performed.

Trial sequential analysis (TSA) was performed with the O'Brien‐Fleming alpha‐spending function for estimating group sequential boundaries utilizing TSA software version 0.9.5.10 Beta (Copenhagen Trial Unit, Centre for Clinical Intervention Research). TSA was used to define boundaries of statistical significance to help determine when future meta‐analysis can be stopped early for conclusive evidence with regard to ideal sample size.

### 2.8 Subgroup Analyses

To evaluate potential sources of heterogeneity, subgroup analysis for non‐trauma laparotomy cases by type of operation (namely open compared to laparoscopic repair) as well as recurrence outcomes related to mesh characteristics (intra‐peritoneal compared to extra‐peritoneal and non‐absorbable compared to absorbable) was performed where possible.

## 3 Results

### 3.1 Literature Search

**FIGURE 1:**
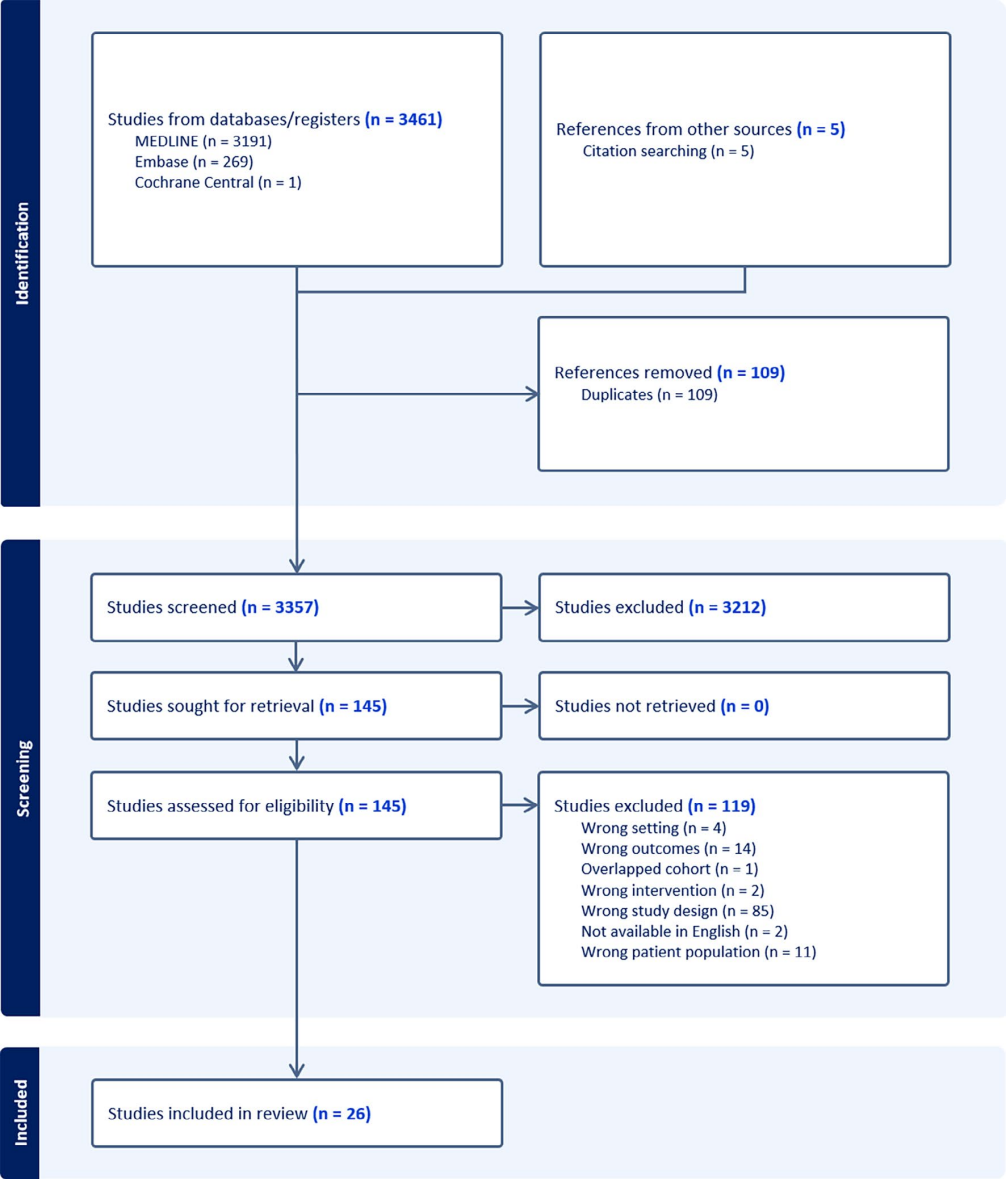
PRISMA flowchart demonstrating flow of literature search, screening and selection of eligible articles.

The initial literature search identified 3357 unique articles for title and abstract screening. Of these, 145 articles progressed to full‐text review, with 119 excluded based on eligibility criteria. Twenty six articles were included in the final analysis. The PRISMA flowchart is shown in Figure 1.

### 3.2 Description of Included Studies

Study characteristics and patient demographic data are presented in Table 1. The 26 studies included a total of 664 patients [2, 3, 4, 5, 6, 7, 8, 13, 14, 15, 16, 17, 18, 19, 20, 21, 22, 23, 24, 25, 26, 27, 28, 29, 30, 31]. Of these, 432 patients underwent operative TAWH repair and therefore were included in the quantitative analysis. Studies were published between 1973 and 2022. The most frequent study type were case series (*n* = 13, 50%) followed by observational retrospective cohort studies (*n* = 12, 46%). Sample sizes in the studies ranged from 2 to 281 patients, with 2 to 176 patients undergoing operative repair of a TAWH. There was inconsistent and poor reporting of time to recurrence, with only 9 of 26 articles reporting on this parameter. Of these, time to recurrence was highly variable and ranged from within the first 24 h to 7 years. Demographic factors including age, location, and management of hernia were unable to be pooled due to a lack of homogeneous data and absence of reporting in some studies. All studies evaluated the impact of acute compared to elective TAWH repair with reporting of recurrence rates.

**TABLE 1:** Overview of included studies.

Author	Year	Country of publication	Study design	Age	Sex	Total patients	BMI	ISS	Location of hernia	Time to recurrence	Acute recurrence	Acute repairs	Elective recurrence	Elective repairs
Abo‐Alhassan	2022	France	Retrospective cohort study	41.75 (11–85)	M: 5, F: 3	8	NR	Not provided however grade of TAWH (per Dennis et al.) provided with only severe cases (IV, V, VI) included	Right‐sided: 4, Flank: 1, Inguinal: 1, Midline: 1, Other: 1 (Multiple)	7 years	1	7	0	0
Agarwal	2009	India	Case series	39 (38–40)	M: 1, F: 1	2	NR	NR	RLQ: 1, Anterior: 1	N/A	0	2	0	0
Akbaba	2015	India	Case series	48.5 (41–56)	2	2	NR	Not provided but 4 patients had bowel injury, 1 patient had solid organ injury, 3 had no injuries and 1 had a muscle injury	LLQ: 1, RIF: 1	N/A	0	0	0	2
Belgers	2005	Netherlands	Case series	54 (44–64)	M: 2, F: 0	2	NR	NR	Left flank: 1, LLQ: 1	N/A	0	2	0	0
Bender	2007	USA	Prospective cohort study	36.4 (13–66)	M: 10, F: 15	25	NR	34.6 (6–16)	Flank: 25	NR	2	11	1	12
Brenneman	1995	Canada	Retrospective cohort study	32 (19–49)	M: 7, F: 2	9	NR	25.3+/−8.1	Flank: 4, Anterior lower abdomen: 3, Mid‐anterior: 2	NR	0	2	2	5
Burt	2004	USA	Case series	41.6 (32–48)	M: 2, F: 1	3	NR	NR	Lumbar: 3	6 months	0	0	1	3
Chow	2020	USA	Retrospective cohort study	31+/−11	M: 8, F: 7	15	33.4+/−7.1	15 +/− 9	Ventral: 7, Lumbar: 8	NR	4	14	0	1
Coleman	2016	USA	Retrospective cohort study	38.6 (9–81)	M: 50, F: 30	80 (23 underwent repair)	NR	22 (17–31)	Anterior: 13, Flank: 36, Inguinal: 3, Lumbar: 25, Spigelian 3, Other: 5 (anterior + flank: 4; lumbar + flank 1)	NR	4	18	2	5
Damschen	1994	USA	Case series	37.2 (5–72)	M: 3, F: 2	5 (3 underwent repair)	NR	NR	RLQ: 1, LLQ: 3, Flank: 1	7 years	1	2	0	0
Danto	1976	USA	Case series	28.3 (24–33)	M: 2, F: 1	3	NR	NR	Anterior: 3	N/A	0	3	0	0
Fullerton	1983	USA	Case series	21.5 (19–24)	M: 2, F: 0	2 (1 underwent repair)	NR	NR	Anterior: 2	N/A	0	1	0	0
Guly	1983	UK	Case series	41.5 (38–45)	M: 2, F: 0	2	NR	NR	LUQ: 1, Inguinal: 1	N/A	0	1	0	1
Gutteridge	2014	Australia	Case series	36.8 (20–59)	M: 3, F: 2	5 (4 underwent repair)	NR	NR	Rectus: 2, RLQ: 1, LLQ: 1, intercostal: 1	N/A	0	1	0	3
Harrell	2021	USA	Retrospective cohort study	38.65 ± 18.06	M: 160 (103 underwent repair), F: 121 (73 underwent repair)	281 (176 underwent repair)	29.4+/−7.13	19.41 +/− 12.66	Rectus: 71, Flank: 86, Lumbar: 18, Other: 1	NR	18	157	3	19
Honaker	2014	USA	Retrospective cohort study	35.8 ± 20.1	M: 23, F: 15	38 (30 underwent repair)	NR	17 (1–66)	Lumbar: 25, Other: 13	NR but all within 5 years	3	27	0	3
Karhof	2019	Netherlands	Retrospective cohort study	44 (24–70)	M: 14, F: 7	21 (17 underwent repair)	25 (21–51)	25 (9–59)	Flank: 14, Rectus: 2, Whole abdomen: 1, Other: 4	NR	5	14	0	3
Kumar	2004	India	Case series	27.5 (25–30)	M: 2, F: 0	2	NR	NR	Anterior: 2	N/A	0	2	0	0
Liesegang	2022	Australia	Retrospective cohort study	36 (18–59)	M: 10, F: 16	16 (13 underwent repair)	NR	19.5 (5–34)	Right lateral: 6, Unilateral anterior: 3, Bilateral anterior: 3, Left lateral: 2, Bilateral lateral: 1, Lumbar: 1	NR but within 137 days	1	11	0	2
Netto	2006	Canada	Retrospective cohort study	39.0 ± 12.0	M: 19, F: 15	34 (10 underwent repair)	NR	31 ± 13	Anterior: 2, Posterior: 32 (inferior lumbar triangle: 30, superior lumbar triangle: 2)	3 cases: within 48 h, 2 months and NR	3	8	0	2
Pardhan	2016	Australia	Retrospective cohort study	36 (24–54)	M: 30, F: 14	44 (41 underwent repair)	25–30	23 (no IQR provided)	RUQ: 1, LUQ: 3, RLQ: 18, LLQ: 13, RUQ & LUQ: 0, RLQ & LLQ: 9	7.25 months	0	8	3	33
Park	2018	South Korea	Retrospective cohort study	55 (23–71)	M: 5, F: 4	9 (8 underwent repair)	NR	NR	All lumbar	N/A	0	1	0	7
Payne	1973	USA	Case series	49 (38–60)	M: 1, F: 1	2	NR	NR	R Flank: 2	Within 24 h	1	1	0	1
Singal	2011	India	Case series	50 (45–60)	M: 2, F: 1	3	NR	NR	Anterior abdomen: 3	N/A	0	3	0	0
Vijayalakshmi	2018	India	Case series	35.5 (21–60)	M: 4, F: 0	4	NR	NR	RLQ: 2, Lumbar: 2	N/A	0	4	0	0
Wong	2022	Australia	Retrospective cohort study	44.1 (29.2–59.1)	M: 14, F: 16	47 (30 underwent repair)	NR	29 (20.5–37.5) [24 (15.5–32.5) for those operatively managed]	Lateral: 11, Lumbar: 13, Anterior: 6	Variable, earliest within 10 days.	6	23	1	7

### 3.3 Acute Versus Elective Repair

**FIGURE 2:**
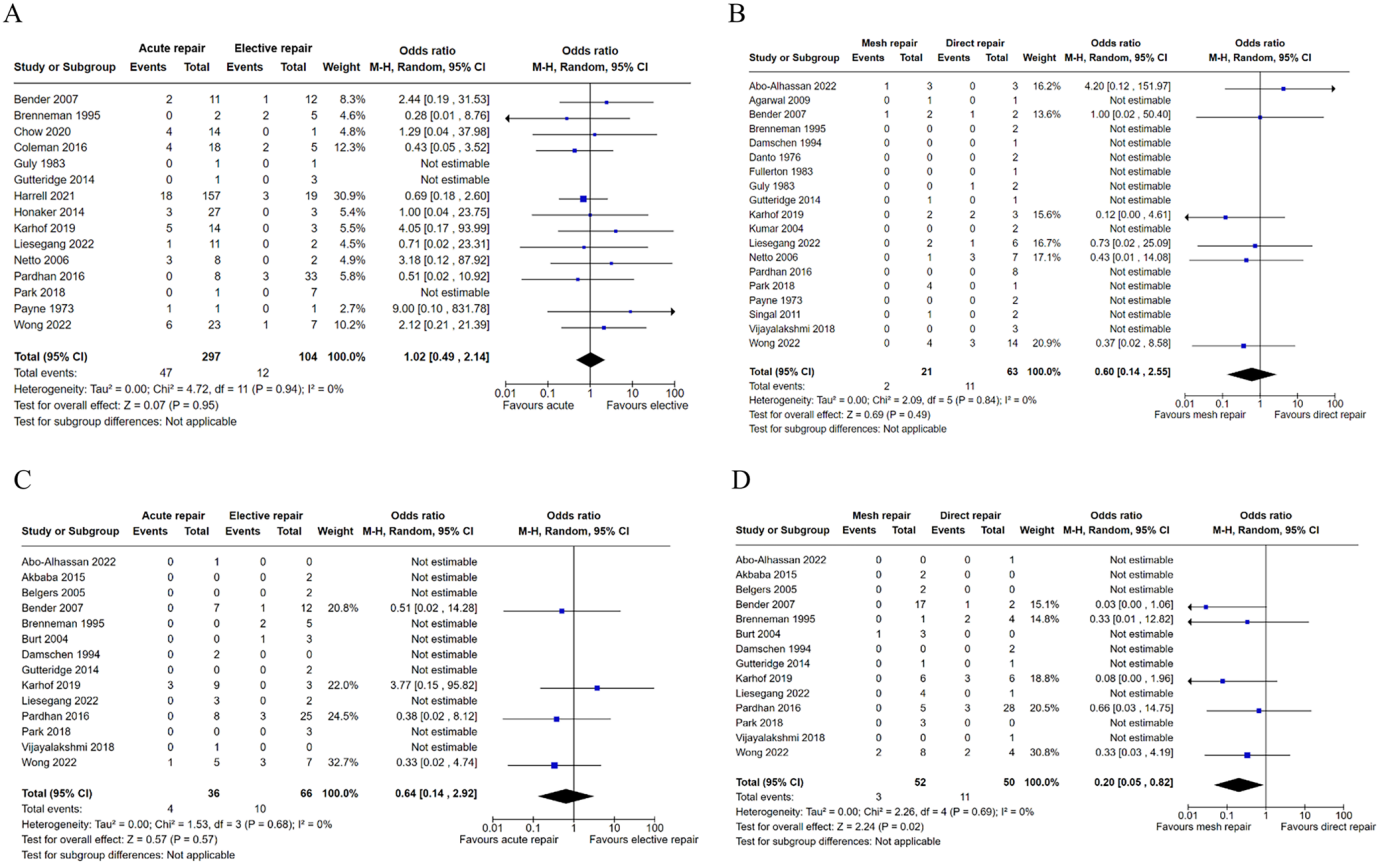
Meta‐analysis of recurrence outcomes in patients with traumatic abdominal wall hernia. (A) Forest plot comparing acute compared to elective repair on recurrence outcomes for all patients underdoing operative repair of traumatic abdominal wall hernia. (B) Forest plot evaluating mesh compared to direct suture repair on recurrence outcomes in patients with traumatic abdominal wall hernia requiring trauma laparotomy. (C) Forest plot evaluating acute compared to elective repair on recurrence outcomes for patients with traumatic abdominal wall hernia who do not require trauma laparotomy. (D) Forest plot evaluating mesh compared to direct suture repair on recurrence outcomes for patients with traumatic abdominal wall hernia who do not require trauma laparotomy.

Hernia recurrence occurred in 49/323 (15.2%) patients who underwent acute repair with or without trauma laparotomy, compared with 13/109 (11.9%) patients who underwent elective repair (Table 1). Pooled analysis of these patients utilizing a random‐effects model revealed no evidence for superiority between acute and elective repair (OR 1.02, 95% CI 0.49–2.14, *p* = 0.95) (Figure 2A).

### 3.4 Mesh Versus Direct Suture Repair in Patients Requiring Trauma Laparotomy

Nineteen studies involving a total of 84 patients evaluated recurrence of TAWH in patients who underwent concurrent trauma laparotomy and repair (Table 2). Mechanisms of injury leading to emergency laparotomy were heterogeneous and included motor vehicle crash, crush injuries, and high falls. Indications for laparotomy were predominantly due to hemodynamic instability or concurrent injury to abdominal viscera. Of the 84 patients in this pooled cohort, hernia recurrence occurred in 2/21 (9.5%) patients who underwent mesh repair and in 11/63 (17.5%) patients who underwent direct suture repair. Pooled analysis of these patients utilizing a random‐effects model showed no evidence for a difference between the groups (OR 0.60, 95% CI 0.14–2.55, *p* = 0.49) (Figure 2B). Reasons for opting out of mesh repair in this population included presence of contamination and size of abdominal wall defect. In some studies, suture repair was considered the only option of repair.

**TABLE 2:** Recurrence outcomes comparing mesh to direct suture repair in patients with traumatic abdominal wall hernia who underwent trauma laparotomy.

Author	Year	Mesh repair recurrence	Mesh repair total	Direct repair recurrence	Direct repair total	Indication for laparotomy	Degree of contamination	Need for further laparotomy	Mesh placement	Follow‐up
Abo‐Alhassan	2022	1	3	0	3	Case 3: evisceration of bowel with hollow viscus injury (TI injury) and hemoperitoneum without significant contamination. Case 4: abdominal stab wounds with eviscerated bowel and multiple SB enterotomies. Case 5: Grade 3 liver with active bleeding, no hollow viscus injury. Case 6. Abdominal gunshot wound to left flank with free fluid and pneumoperitoneum. Case 7: hemoperitoneum following blunt trauma from MVA and unstable Case 8: Hemoperitoneum secondary to fall from 6 m with grade 5 liver requiring pre‐operative embolisation.	Case 3: minimal, Case 4: minimal except for AL, Case 5: minimal. Case 6: minimal. Case 7: minimal Case 8: minimal	Case 3: nil, Case 4: yes due to AL from SB resection, Case 5: yes, for pneumoperitoneum secondary to sigmoid perforation. Case 6: nil Case 7: yes, 48 h post DCL Case 8: yes, DCL followed by second laparotomy 48 h later	Case 3. Vicryl mesh placed intraperitoneal to augment primary closure due to tissue loss. Case 4. Biosynthetic mesh placed at second laparotomy (first was damage control). Anastomotic leak occurred lading to mesh removal and replacement with vicryl intraperitoneal mesh. Hernia recurred Case 5: Intraperitoneal vicryl mesh repair in index laparotomy, not explanted in subsequent laparotomy. Case 6: nil, Case 7: Nil Case 8: nil	Of 8 patients: 5 had follow‐up from 1 to 7 years. 1 had follow‐up to 4 months and 1 had no information on follow‐up
Agarwal	2009	0	1	0	1	Case 1: Closed defect to lower abdomen following bull horn injury in a stable patient. Case 2: evisceration of bowel with intra‐abdominal free fluid following MVA	Case 1: Nil Case 2: Minimal	Case 1: Nil Case 2: Nil	Case 1: preperitoneal mesh of unclear type used Case 2: nil	Of 2 patients, 1 patient was followed up 1‐month post‐op and 1 patient 1 year post‐op
Bender	2007	1	2	1	2	Associated injuries (bowel = 6, genitourinary, spleen = 5, aorta = 1)	NR	Due to concurrent injury including SB injury	2 had absorbable mesh at 2nd or third relook	NR
Brenneman	1995	0	0	0	2	Concurrent injury including to rectosigmoid [4], diaphragm [1], SB perforation [1], spleen rupture [1] liver laceration [1]. 7 had trauma laparotomy but only 2 had repairs attempted and completed	NR	NR	Nil	NR
Damschen	1994	0	0	0	1	Case 1: MVA with shock and 4 quadrant blood on DPL, laparotomy showing ruptured ileum and herniation of SB through LLQ TAWH	Case 1: not reported but had 4 quadrant blood	Case 1: Nil	Case 1: Nil	7 years
Danto	1976	0	0	0	2	Case 1: Patient crushed by airplane horizontal stabilizer transversely across abdomen without open wounds. Bruising noted linearly across abdomen from lank to flank, moderate guarding but no peritonism. Defect felt at bruising site. Case 2: bridge beam crush injury to mid abdomen with bruising across central abdomen linearly and marked tenderness in lumbar area and right flank. Laparotomy showed avulsed right colon and tearing of SB mesentery in multiple places. Patient passed away so TAWH not repaired Case 3: MVA wearing seatbelt at 8‐ miles per hour, linear bruising across abdomen from iliac crest to contralateral iliac crest, peritonism. Crush injury to SB with avulsion and perforation of caecum and avulsion of sigmoid + contamination	Case 1: Hemoperitoneum with crush injury to ileum with degree of intestinal contamination to skin Case 2: Contaminated Case 3: Contamination	Case 1: Repair of TAWH but skin left open due to intestinal contamination which was closed in a weeks' time Case 2: N/A due to patient death Case 3: Repair occurred at index laparotomy, however patient returned for aortic resection a few weeks post index laparotomy in the context of an abdominal thrill and bruit being heard pre‐index laparotomy	Case 1: Nil Case 2: N/A (not repaired in context of death) Case 3: Nil	NR
Fullerton	1983	0	0	0	1	Case 1: MVA, found at scene significant distance from vehicle, agitated on arrival, large mass in right inguinal region which was peristaltic, absent femoral pulses on same side beneath mass and perineal wound (not involving rectum), shocked with no response to 8 units of red cells. Avulsion of hepatic flexure mesentery, caecum infarcted, ascending colon infarcted. Defect too large and patient unwell, left open, patient died so hernia not repaired Case 2: Pinned between 15 t truck and wall of loading dock, abdominal pain on right side with a new bulge, peritonitic, DPL has gross blood. Laparotomy found mid jejunal avulsion form mesentery, retroperitoneal haematoma. Treated and primary closure of defect	Case 1: Contaminated Case 2: Minimal	Case 1: Planned for 2nd laparotomy however died prior Case 2: Nil	Case 1: N/A, died prior to repair Case 2: Nil as primary closure	NR
Guly	1983	0	0	1	2	Case 1: MBA vs. stationary car without peritonism, noted day 10 to have lump overlying left chest wall which contained bowel. Laparotomy performed on day 45 when stabilized. Case 2: Wall collapsed onto patient who was then buried to level of umbilicus while digging a trench. Blunt injury to right groin area with new traumatic mass. Laparotomy a few hours post injury and primary repair of traumatic inguinal hernia which recurred 4 months later	Case 1: Nil Case 2: Nil	Case 1: Nil Case 2: Nil	Case 1: Nil Case 2: Nil	5 months to 2 years
Gutteridge	2014	0	1	0	1	Case 1: MVA with right rectus TAWH managed with repair on second laparotomy (4 days post initial DCL). Sutured closure Case 2: Lower anterior abdominal wall disruption post MVA (car v brick wall) with evisceration of abdominal contents, pneumoperitoneum and free fluid int. he abdomen. DCL—Sections of terminal ileum, mid‐jejunum and sigmoid colon were necrotic secondary to mesenteric avulsion, multiple resections performed. Defect repaired from re‐look laparotomy on Day 2 with definitive closure with mesh on day 20	Case 1: Nil Case 2: NR	Case 1: Repair could not occur in first (laparotomy (DCL) due to size of defect so was returned to ICU for 4 days and definitely repaired post) Case 2: Multiple repeat laparotomies	Case 1: Nil Case 2: Composix sutured to rectus with loop PDS, then a few days later vicryl netted mesh was placed for definitive closure but unclear location	3–5 months
Karhof	2019	0	2	2	3	5 underwent trauma laparotomy, 3 had peritoneal contamination, distribution of other injuries included 38% bowel injury, 10% liver injury, 10% spleen injury, 10% kidney injury, 38* pelvic injury, 43% spinal injury but no specific information about counts related to laparotomy	3 had peritoneal contamination. It is unclear whether they all had direct repair or some had mesh	NR	Depending on the size of the defect, as well as the possible contamination, the choice whether or not to use a mesh was made during surgery by the individual surgeon. Small defects or when there was obvious contamination, were primarily repaired. When meshes were used they were all placed extraperitoneal retromuscular augmenting the repair of the muscular defect in this way. If transmuscular fixation of the mesh could not be obtained, the mesh was fixated to surrounding bony structures such as the iliac crest or the superior ramus of the pubic bone, using anchors or sutures through drilled holes. Type of mesh not provided	17 months (2–60 months)
Kumar	2004	0	0	0	2	Case 1: hit by bus with left anterior abdominal wall swelling. Laparotomy showed no intraabdominal injuries Case 2: e‐scooter vs. parked bus with new swelling over epigastrium with no intra‐abdominal injury	**Case 1: Nil Case 2: Nil**	Case 1: Nil Case 2: Nil	Case 1: Nil Case 2: Nil	4 months to 1 year
Liesegang	2022	0	2	1	6	9 underwent trauma laparotomy with 8 having evidence of TAWH (within 24 h). 4 had SB injury (bucket handle mesenteric injury: 3, jejunal perforation: 1) and underwent SB resection, anastomosis, primary repair without mesh, 2 had colonic injury (rectosigmoid seromuscular tear: 1, sigmoid mesentery bucket handle injury: 1) and underwent oversewing of defect with mesh repair and Hartmann's with primary repair respectively and 1 had intraperitoneal bladder rupture and underwent bladder repair without TAWH being repaired. 2 had no injuries on laparotomy with repair of TAWH (mesh: 1, primary: 1)	5 of seven patients who underwent direct repair had dirty/contaminated/clean contaminated injuries. of 6 patients who underwent mesh repair had dirty/contaminated/clean contaminated injuries	Not reported, all cases had hernias repaired at index laparotomy	Overall, 3 had vicryl mesh, 1 had BIO‐A mesh, 1 had Ventralex ST mesh and 1 had unknown mesh placed	55.5 days (18–137)
Netto	2006	0	1	3	7	8 patients had acute hernia repair with laparotomy	NR	Suture repair was performed because of concomitant bowel contamination	Nonabsorbable mesh, area of placement not reported	Up to 16 months however 11 patients of 34 lost to follow‐up
Pardhan	2016	0	0	0	8	Pelvic fractures (29/44; 66%) were the commonest concomitant injuries followed by thoracic (25/44; 57%) and intra‐abdominal (24/44; 55%) injuries. 12/47 patients had grade VI hernias with herniation of abdominal contents. Eight (20%) operations were carried out on an emergent basis due to the presence of a simultaneous bowel perforation. Five of these patients had both small and large bowel injuries; one had small bowel perforations alone, and two had large bowel injuries (both left colon). The	NR	NR	Nil as all laparotomies had primary repair (mesh repair only in elective cases)	1 to 51 months with an average time of 16 months.
Park	2018	0	4	0	1	Concurrent injury including ascending colon perforation	Variable	Nil	In the open repair technique, a prosthetic mesh was applied in 4 patients, except in 1 patient with an ascending colon perforation. Onlay or sublay mesh placement with transfascial fixation was used. In 1 open case, we used a titanium tacker to fix the prosthetic mesh on the fascia layer for stability.	6 months
Payne	1973	0	0	0	2	Case 1: MVA with seatbelt bruise and positive DPL and peritonism. Case 2: Head on collision MVA into railroad barrier rails with significant pain intrusion, abdominal bruising, haematuria and blood from NG output.	Case 1: hemoperitoneum but minimal contamination Case 2: Minimal	Case 1: Delayed repair on follow‐up Case 2: second laparotomy 24 h post initial with repair of TAWH	Case 1: Nil Case 2: Nil	NR
Singal	2011	0	1	0	2	Case 1: Obstipation following blunt abdominal trauma secondary to fall from a cart with superficial abdominal bruising but not peritonitic. Case 2: pain and constipation following fall from height onto object with blunt trauma to abdomen and hernia on CT (closed) and an ileal perforation. Case 3: gored by a cow with abdominal pain, swelling and bruising to right lower quadrant	Case 1: Nil Case 2: Minimal Case 3: nil	Case 1: Nil Case 2: Nil Case 3: Nil	Case 1: onlay prolene mesh, no contamination Case 2: nil Case 3: nil	3–6 months for 2 patients with 1 patient follow‐up unclear in terms of duration
Vijayalakshmi	2018	0	0	0	3	Case 2: fall from two‐wheeler with abdominal impact and known bowel herniation (closed) on clinical exam and CT. Peritonitic. Mesenteric tear noted. Case 3: MVA blunt trauma to abdomen with new swelling (identified on CT to be lumbar hernia). Case 4: fall from two‐wheeler with blunt abdominal injury and new abdominal swelling	Case 2: minimal Case 3: minimal Case 4: minimal	Case 2: Nil Case 3: Nil Case 4: nil	Case 2: Nil Case 3: nil Case 4: Nil	10 days to 6 months
Wong	2022	0	4	3	14	20 patients underwent trauma laparotomy. The intra‐abdominal injuries included small bowel 15 (32%), colonic 13 (28%), mesenteric 5 (11%), diaphragmatic 3 (6%), spleen 4 (8%), ovary 1 (2%), renal tract 1 (2%), and vascular 1 (2%) (aortic and SMA).	NR	Re‐look in 1 followed by extraperitoneal biomesh repair. Relook in 1 followed by direct repair. 1 had preperitoneal mesh repair on second laparotomy. Relook in one following laparostomy with extraperitoneal biomesh repair. 1 had preperitoneal biomesh following 3 laparotomies. 1 had direct repair following 2 laparotomies. 1 had repair on second laparotomy. 1 had direct repair on second laparotomy	1× pre‐peritoneal BioA mesh. 1× extra‐peritoneal biomesh repair. 1× pre‐peritoneal mesh. 1× extraperieontal biomesh. 1× preperitoneal BioA mesh	12 months (IQR 4–20) with no data available for 1 patient out of 30

### 3.5 Acute Versus Elective Repair and Mesh Versus Direct Suture Repair in Patients Who Did Not Undergo Trauma Laparotomy

Fourteen studies involving a total of 102 patients evaluated recurrence of TAWH in patients who did not undergo trauma laparotomy. These studies evaluated recurrence in the context of acute versus elective repair (Table 3) and mesh versus direct suture repair (Table 4).

**TABLE 3:** Recurrence outcomes evaluating timing of repair in patients with traumatic abdominal wall hernia who do not require trauma laparotomy.

Author	Year	Acute repair recurrence	Acute repair total	Elective repair recurrence	Elective repair total
Abo‐Alhassan	2022	0	1	0	0
Akbaba	2015	0	0	0	2
Belgers	2005	0	0	0	2
Bender	2007	0	7	1	12
Brenneman	1995	0	0	2	5
Burt	2004	0	0	1	3
Damschen	1994	0	2	0	0
Gutteridge	2014	0	0	0	2
Karhof	2019	3	9	0	3
Liesegang	2022	0	3	0	2
Pardhan	2016	0	8	3	25
Park	2018	0	0	0	3
Vijayalakshmi	2018	0	1	0	0
Wong	2022	1	5	3	7

**TABLE 4:** Recurrence outcomes evaluating mesh compared to direct suture repair in patients with traumatic abdominal wall hernia who do not require trauma laparotomy.

Author	Year	Mesh repair recurrence	Mesh repair total	Direct repair recurrence	Direct repair total
Abo‐Alhassan	2022	0	0	0	1
Akbaba	2015	0	2	0	0
Belgers	2005	0	2	0	0
Bender	2007	0	17	1	2
Brenneman	1995	0	1	2	4
Burt	2004	1	3	0	0
Damschen	1994	0	0	0	2
Gutteridge	2014	0	1	0	1
Karhof	2019	0	6	3	6
Liesegang	2022	0	4	0	1
Pardhan	2016	0	5	3	28
Park	2018	0	3	0	0
Vijayalakshmi	2018	0	0	0	1
Wong	2022	2	8	2	4

With respect to the timing of repair, hernia recurrence occurred in 4/36 (11.1%) patients who underwent acute repair and in 10/66 (15.2%) patients who underwent elective repair. Pooled analysis of these patients utilizing a random‐effects model showed minimal evidence for a difference between groups (OR 0.64, 95% CI 0.14–2.92, *p* = 0.57) (Figure 2C).

When evaluating the same population with respect to mesh versus direct suture repair, hernia recurrence occurred in 3/52 (3.9%) patients who underwent mesh repair and in 11/50 (22.0%) patients who underwent direct sutured repair. Pooled analysis of these patients utilizing a random‐effects model revealed moderate evidence favoring mesh repair (OR 0.20, 95% CI 0.05–0.82, *p* = 0.02) (Figure 2D).

### 3.6 Trial Sequential Analysis

Of the 19 studies evaluating trauma laparotomy cases, TSA was used to estimate the required information size to detect a 30% relative risk reduction of recurrence (considered a clinically relevant difference) in mesh compared to direct suture repair with an alpha of 5% and beta of 20% (equivalent to 80% power). The analysis assumed a 14% control incidence rate (the average recurrence rate within the cohort of this study). A model‐based heterogeneity correction of 10% (*D* ^2^) was applied given these parameters. TSA was inconclusive for this outcome as the boundary TSA was ignored due to insufficient information size (4.18%).

**FIGURE 3:**
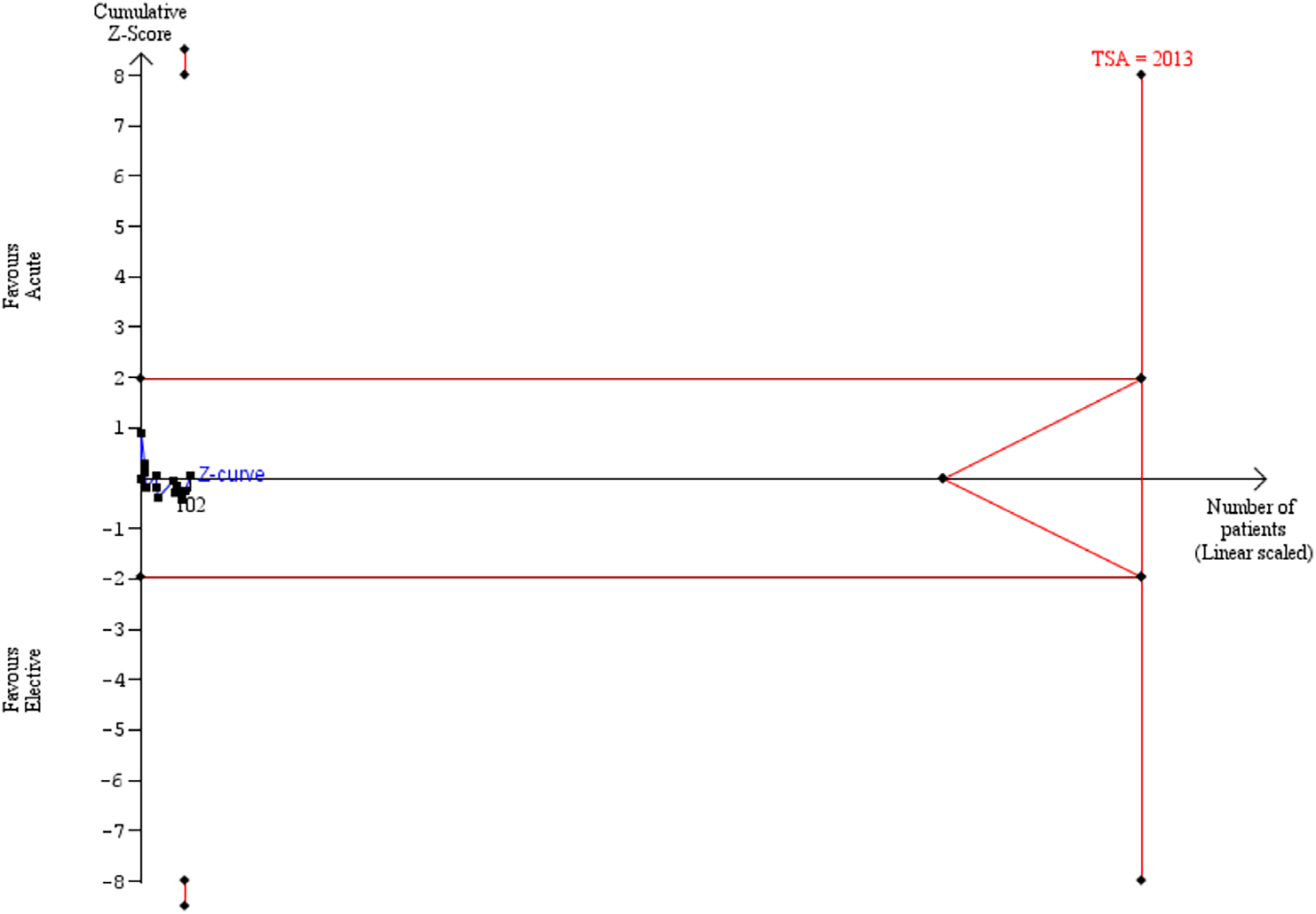
Trial sequential analysis evaluating patients who do not require laparotomy.

TSA evaluating patients who do not require laparotomy was performed to estimate the required information size to detect a 30% relative risk reduction of recurrence when comparing acute to elective repair with the same parameters and a model‐based heterogeneity correction of 10% (*D* ^2^) (Figure 3). The required information size was 2013. TSA was not performed for mesh versus direct suture repair in patients who do not undergo a trauma laparotomy, as the random effects meta‐analysis demonstrated a significant result.

### 3.7 Quality and Risk of Bias Assessment

**FIGURE 4:**
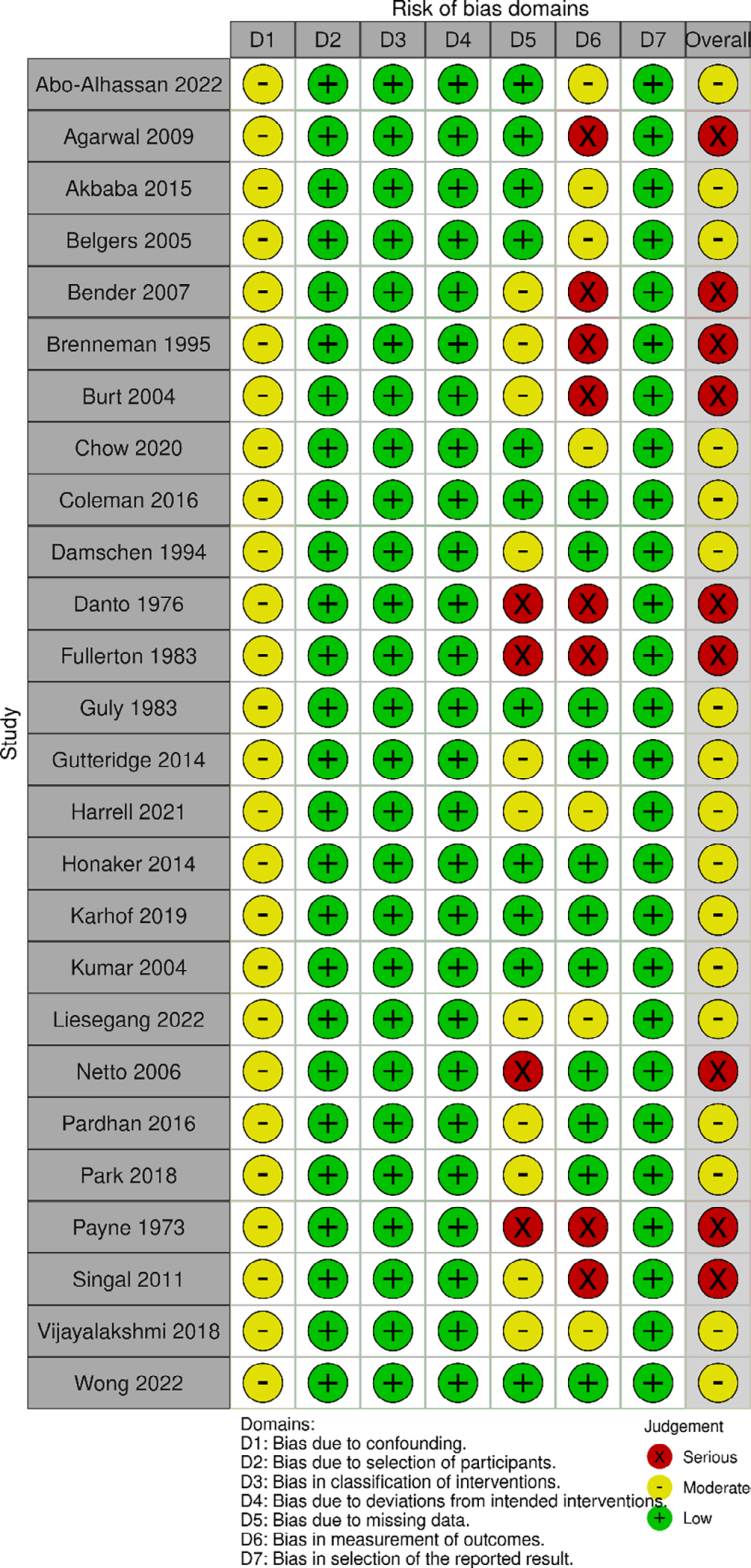
Quality assessment of included articles utilizing the ROBINS‐I assessment tool.

Quality and risk of bias assessment demonstrated low to moderate quality for included studies (Figure 4). Specifically, 17 studies were overall of moderate quality with moderate risk of bias (65.4%), and 9 studies were overall of low quality with serious risk of bias (34.6%). Key areas of bias were lack of control of confounders, small sample size, missing data, and short length of follow‐up.

After removing studies with non‐estimable data, 15 studies were included in funnel plot analysis, which showed no significant qualitative asymmetry; therefore, excluding a high chance of publication bias (Figure S1).

## 4 Discussion

This systematic review and meta‐analysis demonstrates moderate evidence to suggest a reduced odds of recurrence with mesh compared to direct suture repair in patients with TAWH who do not require trauma laparotomy. However, we demonstrate no evidence to suggest superiority for mesh compared to direct suture repair for the management of TAWH in patients requiring trauma laparotomy. Similarly, in patients with TAWH who do not require a trauma laparotomy, there was no evidence to demonstrate superiority in the choice of timing of repair. However, there was noticeably poor reporting of time to recurrence, which was highly variable between studies and within studies was limited by the presence of missing data. Our analysis also highlights, despite the absence of statistical heterogeneity, that there was substantial clinical heterogeneity with respect to included studies leading to a higher risk of bias. Sources for this included the diversity of the population with respect to age, demographics, and study size, the mechanisms of injury, location and severity of TAWH, nature and severity of concurrent visceral injury, degree of contamination, and preferred surgical technique.

The influence of approach to and timing of TAWH repair on hernia recurrence outcomes remains poorly characterized. As such, trauma centers have adopted an array of approaches to management. This may be explained by the relatively low incidence rate of TAWH and the need to manage more urgent and life‐threatening concurrent injuries [2, 3]. There is a preference for acute repair (75% of cases) without mesh in this population. This likely stems from the need to manage concurrent injuries causing abdominal contamination, regional differences in management as the majority of articles were from the United States of America, and surgeon preferences. The latter is a major confounder, as in earlier studies laparoscopy and mesh repair were not viable options for management. Results from larger cohort studies included in this review have yet to yield significant results when comparing acute and elective repair [5, 8]. However, currently the rationale for early repair in patients not requiring trauma slaparotomy remains unclear. In particular, Wong and colleagues identified that acute repair was associated with higher recurrence rates, which raises the question as to the benefit of acute repair at all [5]. In their retrospective study, the authors recommended delayed repair for multiple reasons, including physiological recovery from polytrauma, reduced contamination risk and better scarring or definition of hernia margins to facilitate a more robust reconstruction [5]. This was not found by other retrospective studies [8]. However, for the trauma patient, there are also logistical considerations that must be balanced, such as the social circumstances of the patient, potential for losses to follow‐up, willingness to further engage in healthcare and competing interests of rehabilitation for concurrent injury that may make it difficult to facilitate timely elective repair. In these cases, acute repair may be warranted. From a safety perspective, hernia enlargement and potential incarceration is a theoretical concern that may prompt acute repair. Despite this, Wong et al. identified no cases of acute incarceration in patients with TAWH who underwent non‐operative management (n=17) [5]. In settings where trauma laparotomy was not required, there was a higher proportion of TAWH repairs that used mesh, which were associated with lower rates of recurrence (5.8% vs. 22%); however, the paucity of data limits interpretation of these results. Our data however mirrors recommendations of evidence‐informed algorithms in the literature. For example, Wong and colleagues suggest that mesh repair be used for patients who do not require trauma laparotomy and are amenable to elective repair [5]. Furthermore, for patients who require a trauma laparotomy, and with risk of contamination, primary suture repair is recommended [5]. This is consistent with recommendations made from retrospective observational data from similar trauma centres [4].

Given the heterogeneity and non‐significant outcomes identified, we performed a TSA of pooled data to estimate sample size for prospective trials. TSA is generally used in interventional or randomised‐controlled studies; however, there is evidence of its validity for use in observational settings [32]. TSA was inconclusive for recurrence in mesh compared to direct suture repair in patients requiring trauma laparotomy due to small sample size. Optimal information size was 2013 for those who do not require trauma laparotomy with respect to timing of repair. Our results therefore suggest that trials or cohort studies with larger samples may be required. However, it is important to note that trials of this size may not be feasible due to the relative rarity of TAWH. Furthermore, there are likely to be confounding influences with respect to decision making for patients with a TAWH where there may be a preference for acute repair in some institutions due to necessity or a preference to avoid mesh due to concerns related to a contaminated field. To gain more robust evidence, clinicians may have to consider other possible sources of evidence, including international registries for prospective data collection or larger retrospective multi‐center international cohort studies. To further evaluate heterogeneity, we also considered subgroup analysis for non‐trauma laparotomy cases by type of operation (namely open compared to laparoscopic repair) as well as recurrence outcomes related to mesh characteristics (intra‐peritoneal compared to extra‐peritoneal and non‐absorbable compared to absorbable); however, there was insufficient information to perform these analyses despite attempts to contact corresponding authors for unpublished data.

There are several limitations to note within this review. The quality of current evidence arises predominantly from retrospective observational studies of low or moderate methodological quality. In addition to the small sample sizes, there is a lack of control and standardization for confounders, including patient factors such as smoking, body mass index, presence of diabetes, degree of injury (injury severity score, degree of contamination) and presence of concurrent pre‐existing abdominal hernias at the time of TAWH diagnosis. There was also poor reporting of other clinically relevant outcomes such as the grade of TAWH, which meant analyses related to recurrence with respect to the severity of TAWH were not possible. Furthermore, included studies were of moderate to high risk of bias attributable to missing data and inadequate follow‐up. This is particularly important as small changes may significantly impact the effect sizes that we have calculated in the TSA. Additionally, given the eligibility criteria of this review only included English language articles, there is the potential for missing data from literature in other languages. Lastly, given there was often inconsistent reporting of demographic data within the included articles, it was not possible to control for confounding demographic factors in the analysis of outcomes within this review.

## 5 Conclusion

This systematic review and meta‐analysis suggests there is evidence that in patients with TAWH who do not require trauma laparotomy, mesh repair is favorable in terms of recurrent outcomes compared to direct suture repair. There was insufficient evidence to favor acute compared with delayed repair, irrespective of whether patients underwent trauma laparotomy or not. This evidence is reported on the background of predominantly retrospective observational studies of moderate to high risk of bias and therefore the true impact of the approach to TAWH repair remains poorly characterized.

## Author Contributions

**Khang Duy Ricky Le:** conceptualization, data curation, formal analysis, investigation, methodology, project administration, resources, validation, visualization, writing – original draft, writing – review and editing. **Su Jin Lee:** data curation, formal analysis, investigation, methodology, resources, validation, visualization, writing – original draft, writing – review and editing. **Rose Shakerian:** formal analysis, investigation, methodology, project administration, supervision, validation, writing – original draft, writing – review and editing. **Benjamin P. T. Loveday:** conceptualization, formal analysis, investigation, methodology, project administration, supervision, validation, writing – original draft, writing – review and editing. **David Read:** conceptualization, formal analysis, investigation, methodology, project administration, supervision, validation, writing – original draft, writing – review and editing.

## Disclosure

A/Prof Benjamin PT Loveday is an Editorial Board member of ANZ Journal of Surgery and a co‐author of this article. To minimize bias, they were excluded from all editorial decision‐making related to the acceptance of this article for publication.

## Conflicts of Interest

The authors declare no conflicts of interest.

## Supporting information


**Figure S1.** Publication bias analysis of included studies using funnel plot.
