# Supplementary material for: Comparative Outcomes of Direct Versus Mesh Repair and Timing of Repair for Traumatic Abdominal Wall Hernias: A Systematic Review and Meta‐Analysis
Source: ANZ J Surg. 2025 Jul 25;95(9):1694–717. doi: 10.1111/ans.70265 (PMC12484395; doi:10.1111/ans.70265)

**Supplementary figure legends**

Supplementary figure 1. Publication bias analysis of included studies using funnel plot.

**Supplementary figures**


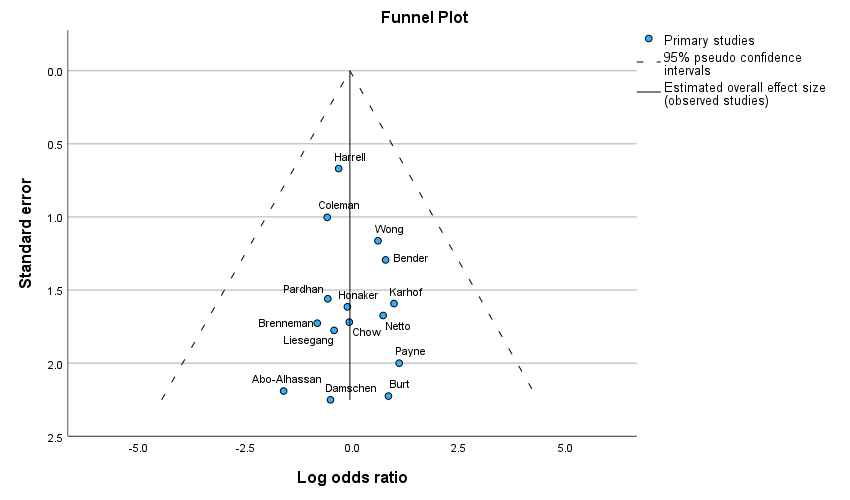

Supplement: Supplementary file 1 — Figure S1. Publication bias analysis of included studies using funnel plot. [file ANS-95-1694-s001.docx]
